# Low-dose esketamine with sufentanil for postcesarean analgesia in women with gestational diabetes mellitus: a prospective, randomized, double-blind study

**DOI:** 10.3389/fendo.2023.1202734

**Published:** 2023-08-11

**Authors:** Tao Han, Qin Chen, Jie Huang, Jie Zhang, Aiyuan Li, Wei Xu, Zheming Peng, Zhen Li, Liang Chen

**Affiliations:** Department of Anesthesiology, Hunan Provincial Maternal and Child Health Care Hospital, Changsha, China

**Keywords:** gestational diabetes, cesarean delivery, intravenous patient-controlled analgesia, esketamine, prospective study

## Abstract

**Background:**

Pregnant women with gestational diabetes mellitus (GDM) require more analgesics after cesarean delivery than those who do not have GDM. Uncontrolled pain following cesarean delivery is a major problem in women with GDM. We investigate the efficacy of low-dose esketamine combined with sufentanil intravenous patient-controlled analgesia (PCA)for postcesarean analgesia in women with GDM.

**Methods:**

One hundred forty pregnant women with GDM were enrolled participate in this randomized controlled trial and were randomized into two groups (70 in each group). The esketamine (S) group was given esketamine +sufentanil + ondansetron, and the control (C) group was given sufentanil +ondansetron. The primary outcome is sufentanil consumption at 24 hours postoperatively, the secondary outcomes are sufentanil consumption at 6 hours postoperatively, pain scores at 6, 24 and 48 hours postoperatively.

**Results:**

Compared with group C, group S had significantly lower sufentanil consumption at 6 and 24 hours postoperatively (P= 0.049 and P<0.001), significantly lower activities VAS(pain during activities)scores at 6 hours postoperatively, rest and activities VAS (pain at rest and pain during activities)scores at 24 hours postoperatively, and activities VAS scores at 48 hours postoperatively(P=0.022, P =0.002, P=0.001 and P=0.007). Compared to group C, the time to bowel function return was significantly shorter in group S. There was no significant difference in rest VAS (pain at rest) scores at 6 and 48 hours postoperatively (P>0.05). The time to first lactation was not significantly different between the two groups (P>0.05). There was no significant difference in neonatal neurobehavioral scores between the two groups (P>0.05).

**Conclusion:**

Compared to sufentanil PCA, adding low dose of esketamine significantly reduced the consumption of sufentanil while providing equally effective post cesarean analgesia in the patients with gestational diabetes.

## 1 Background

GDM is a common complication of pregnancy, and the incidence of GDM rapidly increased in recent years (1). Pregnant women with GDM tend to deliver by cesarean section, and despite the short duration of hyperglycemia in GDM patients, it is still a risk factor for postcesarean pain (2), some studies have found that postcesarean pain is more severe in women with GDM than in those without GDM, and postoperative pain requires more sufentanil to achieve satisfactory analgesia after surgery (3, 4). Clinicians should focus on postoperative analgesic management in patients with GDM to improve the effectiveness of postoperative analgesia.

Esketamine is a noncompetitive antagonist of the N-methyl-D-aspartate receptor (NMDA receptor), which binds to the NMDA receptor and blocks the NMDA receptor from binding to glutamate to exert analgesic and antinociceptive effects (5). In clinical trials (6), esketamine was used as an adjuvant for postoperative pain control, reducing opioid consumption and prolonging the duration of analgesic action.Some studies (7, 8) found that GDM and the onset of postpartum depression are related, that inflammatory pathways are involved in the pathogenesis of GDM-related postpartum depression, and that intravenous patient-controlled esketamine has a preventative effect on postpartum depression (9). Suppa et al. (10) suggested that preoperative use of low-dose esketamine is safe for women, and that intravenous use of low-dose esketamine after cesarean section can reduce maternal morphine use after lumbar neuraxial anesthesia for cesarean section. Esketamine has no adverse effects on uterine blood flow, maternal or fetal hemodynamics (11). Thus esketamine is suitable for postcesarean analgesia in women with GDM. However, more clinical data are still needed to confirm this idea.

We hypothesized that esketamine might produce better analgesia and higher patient satisfaction than conventional anesthetics after cesarean section in women with GDM. To test our hypothesis, we designed this prospective randomized controlled study to evaluate the effect of low-dose esketamine with sufentanil for intravenous patient-controlled analgesia as an analgesic regimen in GDM women.

## 2 Materials and methods

### 2.1 Study design

This prospective study was approved by the Ethics Committee of Hunan Provincial Maternal and Child Health Hospital [No. 2020-S068], and the study participants signed the informed consent form and was conducted between March 1, 2021 and December, 2022.

### 2.2 Population selection criteria

Full-term singleton pregnancies with GDM (those with ASA scores of I-II) were included. Exclusion criteria: (1) patients with ASA scores of III and above; (2) patients with contraindications to neuraxial anesthesia; (3) patients with a history of opioid dependence; (4) patients who were diagnosed as having diabetes mellitus before pregnancy. Withdrawal criteria: (1) any clinical adverse event; (2) severe pain; (3) pregnant woman or family member who were unwilling to complete the study.

### 2.3 Interventions

We screened 150 patients with GDM undergoing elective cesarean section, and then 140 patients were enrolled based on inclusion, exclusion and withdrawal criteria. They were randomly divided into the esketamine (S) group, esketamine 0.5 mg/kg + sufentanil 150ug + ondansetron 4mg and the control (C) group,sufentanil 150ug + ondansetron 4mg, with 70 in each group.

Maternal blood glucose was checked on the morning of the cesarean section. Maternal age, height, weight, gestational week and cesarean section data were recorded. The patients fasted from solid foods for >6 hours and from alcohol for 2 hours before the operation, and no premedication was administrated before the surgery. Patients were educated how to use the PCA pump (FORNIA pump model CPE-101, Zhuhai California Medical Equipment Co., Ltd.) and how to assess pain using a Visual Analog Scale (VAS) before surgery.

After entering the operation room, noninvasive blood pressure (NBP), electrocardiogram (ECG), HR, and SPO2 were standard monitored. Oxygen was administered via nasal catheter at 2L/min, compound sodium chloride was injected. The anesthesiologist adjusted the infusion rate according to the patient’s circulatory status. All patients were placed in the left decubitus position, received CSEA at L3-4 space using the needle-through-needle technique. After the epidural space was identified, a spinal needle was used to puncture the dura mater and enter the subarachnoid space, with 15mg ropivacaine was diluted with cerebrospinal fluid to 2 ml for intrathecal injection, and the epidural catheter was immediately inserted cephalad 3–4 cm. The patients were positioned supine and tilted 15° to the left until fetal extraction. The operation was started when T6 was blocked, and additional ropivacaine can be added to the epidural if necessary. The mean intraoperative blood pressure was maintained at ≥65 mmHg, and if the mean blood pressure fell below 65 mmHg, methoxamine was administered and repeated as needed; in addition, in the case of sinus bradycardia (heart rate <50 bpm), 0.3 mg of intravenous atropine was administered and repeated as needed. After delivery, flurbiprofen ester 50 mg and ondansetron 4 mg are administered intravenously. The epidural catheter was removed at the end of the procedure. The duration of the procedure and the blood loss were recorded. After surgery, use PCA pump. Group S received esketamine 0.5mg/kg + sufentanil 150µg + ondansetron 4mg, and group C received sufentanil 150µg + ondansetron 4 mg, both were diluted to 100ml with saline, the sufentanil concentration was1.5ug/mL, the maintenance dose was 2ml/h, PCA bolus 2 ml at lock out interval of 15 minutes. If the VAS score was ≥7, flurbiprofen was readministered and the patient was excluded from the group. Research assistant who was blinded to the randomization recorded the patients’ PCA pump use (sufentanil consumption), and any adverse effects, such as nausea, vomiting, dizziness, and hallucinatory symptoms. Pain was assessed using the score VAS at 6, 24, and 48 hours postoperatively, with “0 “ indicating no pain and “10 “ indicating the most severe pain imaginable. The time to bowel function return, time to first lactation, and neonatal neurobehavioral scores were recorded.

### 2.4 Outcomes

The primary outcome was sufentanil consumption at 24 hours postoperatively, and secondary outcomes were sufentanil consumption at 6 hours postoperatively, VAS scores at rest and during activities at 6, 24, and 48 hours postoperatively, time to bowel function return(the time to pass flatus as the sign of bowel function return), time to first lactation, adverse effects such as postoperative nausea, vomiting, dizziness, and hallucinations, and neonatal neurobehavioral scores.

### 2.5 Sample size

The primary endpoint of this study was the consumption of sufentanil at 24 hours after surgery. We used a two-tailed test with α = 0.05 and β = 0.1 based on the pre-experimental 24-hour postoperative sufentanil consumption in groups S (95.1 ± 5.5) and C (99.3 ± 7.0), with a minimum of 60 patients required in each group. Ultimately, each group included 75 patients, taking into account a 20% attrition rate.

### 2.6 Randomization and blinding

**Figure 1:**
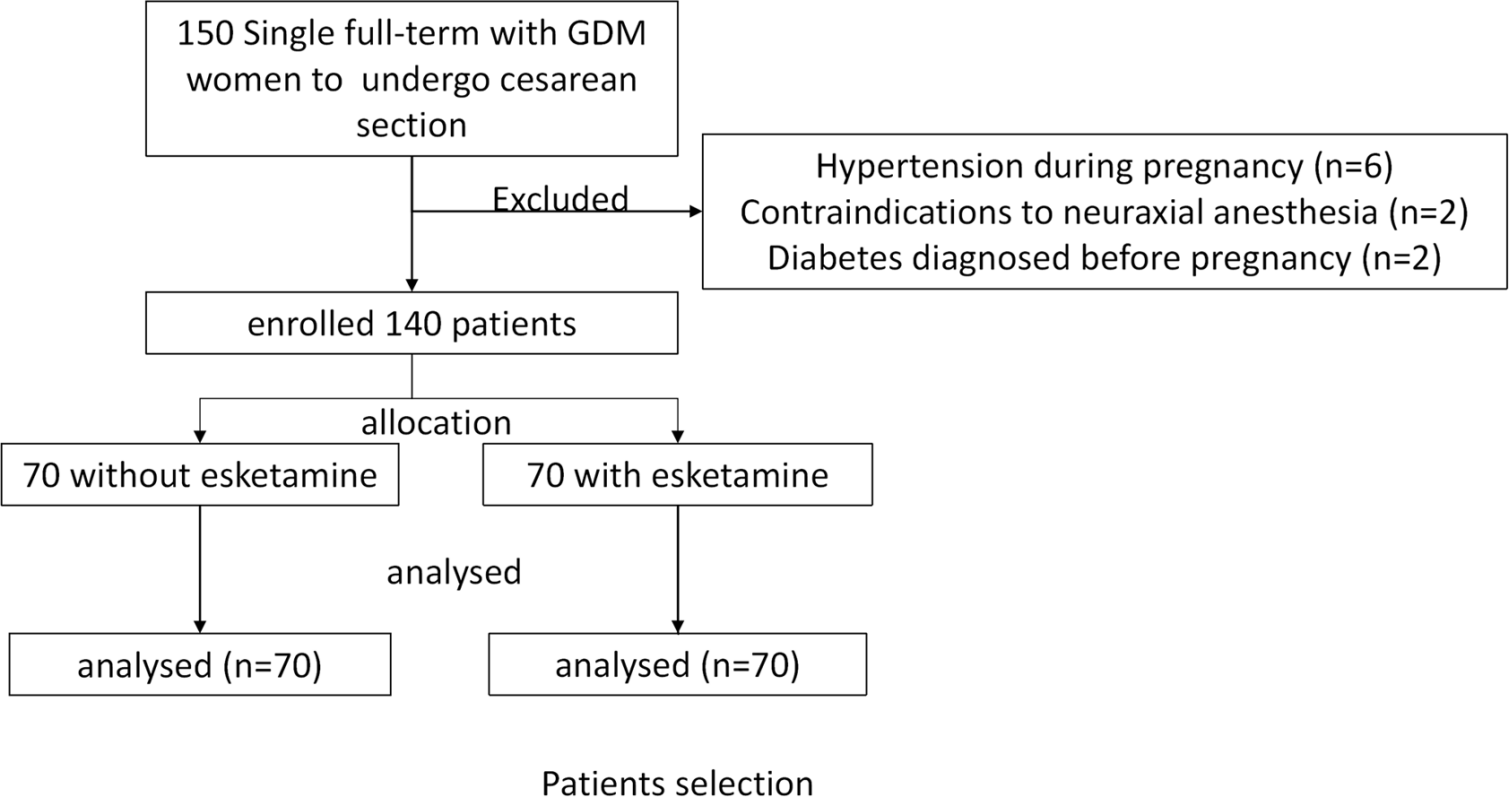
Flow chart of participants.

The investigators used the SAS statistical software package on a computer to generate random numbers in a 1:1 ratio to determine groups S and C. Neither the blinded investigators (the surgeon and the anesthesia resident physician) nor the patients were aware of the study groupings. Before the patients entered the operating room, the attending anesthetist opened the envelope, and the patients were assigned to group S or C according to the randomization entry number. Another anesthetist resident physician did not know the group assignment, and performed the subsequent anesthesia and the postoperative follow-up. A CONSORT diagram shows the participant flow (**Figure 1**).

### 2.7 Statistical analysis

SPSS 25.0 was used for the statistical analysis, and a P value < 0.05 was considered statistically significant. Continuous variables were analyzed with the Mann-Whitney U test or independent samples t test, and the Kolmogorov-Smirnov test was performed first to confirm whether the data were normally distributed. Normally distributed measurements are expressed as the mean ± standard deviation, and non-normally distributed variables are expressed as the median (interquartile range). Categorical variables were compared using the chi-square test or Fisher’s exact test and expressed as percentages. Statistical analyses were performed using SPSS software version 25.0. Two-sided p values less than 0.05 were considered statistically significant.

## 3 Results

A total of 150 women with GDM were screened in this study from March 2021 to December 2022. Based on exclusion criteria, 10 women with GDM were excluded prior to randomization (6 with GDM combined with gestational hypertension, 2 with contraindications to neuraxial anesthesia, and 2 with a prepregnancy diagnosis of diabetes mellitus). A total of 140 women with GDM were included in the study, 70 in each group (**Figure 1**).

### 3.1 Demographic data

There were no significant differences in the basic information of the two groups, including age, height, weight, gestational week, number of cesarean deliveries, preoperative glucose, and duration of surgery (**Table 1**).

**Table 1:** Demographic data.

Variables	Group C	Group S	P-value
	N=70	N=70	
Age, years	32.1 ± 3.9	31.7 ± 3.9	0.511
Duration, mins^†^	65 (55-80)	70 (60-80)	0.199
BMI, kg/m^2^	28.4 ± 3.0	28.0 ± 2.8	0.349
Preoperative blood glucose, mmol/L^†^	5.4 (5.1-6.2)	5.3 (4.9-5.8)	0.459
Gestational week			0.512
37	17 (24.3%)	16 (22.9%)	
38	37 (52.9%)	32 (45.7%)	
39	16 (22.9%)	22 (31.4%)	
Nulliparous			0.175
yes	42 (60.0%)	34 (48.6%)	
no	28 (40.0%)	36 (51.4%)	

^†^median(Q1-Q3).

### 3.2 Comparison of the postoperative analgesic effects between groups

Compared with group C, sufentanil consumption was significantly reduced in group S at 6 and 24 hours postoperatively (P=0.049 and P<0.001),

The activities VAS scores at 6 hours, the rest and activities VAS scores at 24 hours, the activities VAS scores at 48 hours were significantly lower in group S (P=0.022, P=0.002, P=0.001 and P=0.007), with no significant difference in rest VAS scores at 6 hours and at 48 hours postoperatively (**Table 2**).

**Table 2:** Comparison the postoperative analgesic effects between Groups.

Groups	Group C	Group S	P-value
	N=70	N=70	
Sufentanil consumption(P6H), ug^†^	24.0 (24.0-27.0)	24.0 (24.0-26.3)	0.049
Sufentanil consumption(P24H), ug^†^	93.0 (90.0-98.3)	84.0 (81.0-87.0)	<0.001
VAS Rest, P6H^†^	2.0 (2.0-2.8)	2.0 (2.0-2.0)	0.235
VAS Motion, P6H^†^	3.0 (3.0-3.0)	3.0 (3.0-3.0)	0.022
VAS Rest, P24H^†^	3.0 (3.0-4.0)	3.0 (2.0-3.0)	0.002
VAS Motion, P24H^†^	5.0 (5.0-6.0)	5.0 (4.0-5.0)	0.001
VAS Rest, P48H^†^	2.0 (2.0-3.0)	2.0 (2.0-2.0)	0.675
VAS Motion, P48H^†^	3.0 (3.0-4.0)	3.0 (3.0-3.0)	0.007

^†^Median(Q1-Q3); P6H, Postoperative 6h; P24H, Postoperative 24h; P48H, Postoperative 48h;VAS, Visual Analog Scale.

### 3.3 Comparison of the postoperative adverse effects between groups

Compared with group C, the time to bowel function return (P=0.031) was significantly shorter in group S, and there was no significant difference in the time to first lactation (**Table 3**). There was no significant difference in the neonatal neurobehavioral scores between the two groups (**Table 3**).

**Table 3:** Comparison the postoperative adverse effects between Groups.

Groups	Group C	Group S	P-value
	N=70	N=70	
NBNA scores(2 day) ^†^	38 (37- 39)	38 (37- 39)	0.461
Postoperative blood glucose(4h), mmol/L	5.6 ± 0.8	5.4 ± 0.6	0.025
First ambulation, day^†^	2 (2-2)	2 (2-2)	0.319
First lactation, n (%)			0.627
<24h	15 (21.4)	16 (22.9)	
24-48h	43 (61.4)	46 (65.7)	
>48h	12 (17.1)	8 (11.4)	
Bowel function return time, n (%)			0.031
<24h	25 (35.7)	37 (52.9)	
24-48h	29 (41.4)	27 (38.6)	
>48h	16 (22.9)	6 (8.6)	
Nausea, n (%)			0.73
no	65 (92.9)	66 (94.3)	
yes	5 (7.1)	4 (5.7)	
Vomit, n (%)			1
no	67 (95.7)	67 (95.7)	
yes	3 (4.3)	3 (4.3)	
Dazzle, n (%)			1
no	68 (97.1)	68 (97.1)	
yes	2 (2.9)	2 (2.9)	

^†^Median(Q1-Q3); NBNA, neonatal neurobehavioral.

There was no significant difference in the postoperative adverse effects (nausea, vomiting, and dizziness) between the two groups (**Table 3**).

## 4 Discussion

This randomized controlled trial evaluated the postoperative analgesic regimen for GDM women undergoing cesarean delivery. Our results showed that low-dose esketamine combined with sufentanil PCA in group S not only reduced the amount of sufentanil used at 6 and 24 hours postoperatively in GDM women undergoing cesarean delivery, but also significantly lowered the rest VAS scores at 24 hours postoperatively and significantly lowered the activities VAS scores at 6, 24, and 48 hours postoperatively compared with group C.

Many previous studies (12–14) have evaluated esketamine in combination with different opioids by different routes of administration for postoperative analgesia in different surgical types. Han et al. (9) studied the effect of intravenous patient-controlled analgesia with esketamine on postpartum depression and found that esketamine reduced postoperative pain after cesarean delivery. Another recent study found (15) that intraoperative intravenous administration of 0.25 mg/kg esketamine relieved pain during exercise 24 hours after cesarean delivery. In addition, a retrospective study by Ye Wang et al. (16) showed that esketamine controlled pain after cesarean delivery. Although the effectiveness of esketamine combined with opioids for postcesarean analgesia is well established, there are limited data on analgesia in women with GDM who have more severe postcesarean pain than non-GDM women, thus the effect of esketamine in women with GDM is unclear. Our study further evaluated the effect of low-dose esketamine combined with sufentanil intravenous patient-controlled analgesia in GDM women with postcesarean analgesia. A dose of 0.5 mg/kg esketamine for PCA reduced the sufentanil dose, reduced pain scores, and improved analgesia in GDM patients, thereby reflecting the effectiveness of esketamine for pain control in such cases.

Esketamine, the dextroisomer of ketamine, has a higher affinity for NMDA receptors and U-opioid receptors and is used at only 1/2 the dose of ketamine. Previous studies (17) have shown that ketamine at 0.75 mg/kg-1.5 mg/kg did not provide analgesic effects in the biological phase during the postoperative period. Ketamine provided analgesia only at very high doses. One study reported (18), that the effect of small doses of esketamine on NMDA receptors was not considered analgesic but antinociceptive. Animal experiments (19) found that 10 mg/kg ketamine failed to induce an analgesic effect in rats; thus, the response to injurious stimuli was measured by nociceptometry, and although the ketamine dose did not have a sufficient direct anti-injurious effect, it effectively attenuated the development of acute tolerance to alfentanil and inhibited alfentanil-induced nociceptive hyperalgesia. In this experiment, esketamine was administered at a dose of 0.5 mg/kg in an intravenous patient-controlled analgesia pump at an infusion dose of 0.01 mg/kg/h. This dose was not sufficient to provide analgesic effects in the biological phase during the postoperative period, but it increased the analgesic effect while reducing the postoperative sufentanil consumption, which might have been related to its attenuation of opioid analgesic tolerance. Moreover, the effect of esketamine on pain-induced central sensitization might be related to its attenuation of opioid analgesia. The effect of esketamine on pain-induced central sensitization might also be a factor in the reduction in opioid demand. Furthermore, there is a large body of literature (20–22) showing that the use of low-dose ketamine during a cesarean section could enhance analgesia by antagonizing opioid-induced nociceptive sensitization, thereby reducing the use of postoperative morphine. This explains the low opioid requirement in the esketamine group. In addition, it was found that (23), esketamine has anti-inflammatory effects, inhibits pro-inflammatory cytokines, and enhances the production of anti-inflammatory mediators. GDM’s pathological process is an inflammatory response, which worsens women’s pain (24), the anti-inflammatory effect of esketamine, through the inhibition of inflammatory factors, can reduce the development of inflammation associated with GDM postoperatively, and may have a certain effect on the occurrence of pain. But future studies are needed to establish more precise correlations and conclusions regarding the interactions between these drugs.

The presence of hyperglycemia in gestational diabetes patients undergoing surgery had an adverse effect on gastrointestinal function, and this study showed that the proportion of mothers in group S with bowel function return times <24h and 24-48h was significantly higher than that in group C. This result may be related to the fact that esketamine effectively relieved the adverse stress caused by pain, and that esketamine reduced the amount of sufentanil, thus controlling the resulting gastrointestinal adverse effects and facilitating the recovery of bowel function after surgery. However, we need to monitor more indicators related to gastrointestinal function to corroborate this result, and the specific mechanism needs to be further investigated.

Postpartum lactation was mainly the result of the combined action of prolactin and lactogen, and the results of our study showed no significant difference in the time to the first lactation between the two groups. Consistent with the findings of a previous study (10) esketamine exposure did not decrease the patient’s ability to breastfeed or the duration of breastfeeding. There was also no significant difference in neonatal neurobehavioral scores between the two groups, indicating that low-dose esketamine intravenous patient-controlled analgesia did not increase neonatal risk. This was consistent with the 2020 guidelines for anesthesia and sedation for breastfeeding women (25), which categorize ketamine as safe for breastfeeding women. A previous study (10) showing that low-dose esketamine used for postoperative pain management after cesarean section had no effect on the neonate during the observed duration of continuous breastfeeding.

The incidence of nausea, vomiting, dizziness, and hallucinations in this study was not significantly different between the two groups. Consistent with the findings of a previous study (9), the addition of low-dose esketamine to sufentanil intravenous patient-controlled analgesia was well tolerated and did not increase the risk of hallucinations.

This study has some limitations. First, esketamine is contraindicated in patients with severe hypertension and abnormal thyroid function; therefore, the population selected for this study did not include mothers with complicated pregnancies. Second, we did not measure esketamine levels in breast milk, and although studies have reported that low-dose esketamine has no effect on breastmilk or newborns, it is possible to monitor esketamine levels in breast milk in subsequent studies. None of the newborns in this study had any adverse outcomes.

## 5 Conclusion

In conclusion, based on the results from our study, it can be concluded that low-dose esketamine combined with sufentanil for intravenous patient-controlled analgesia can reduce the amount of sufentanil and enhance the analgesic effect after cesarean section in women with GDM, providing new data for the analgesic protocol after cesarean section in women with GDM.

The combination of low-dose esketamine with sufentanil for PCA can enhance the analgesic effect.

## Data availability statement

The raw data supporting the conclusions of this article will be made available by the authors, without undue reservation.

## Ethics statement

The studies involving human participants were reviewed and approved by Hunan Provincial Maternal and Child Health Hospital. The patients/participants provided their written informed consent to participate in this study.

## Author contributions

LC and TH designed the study. TH and QC organized the data. JZ and JH analyzed the data and wrote the first draft of the manuscript. AL, WX, ZL, and ZP revised the manuscript. All the authors contributed to the article and approved the submitted version.
